# Computational modelling of self-reported dietary carbohydrate intake on glucose concentrations in patients undergoing Roux-en-Y gastric bypass versus one-anastomosis gastric bypass

**DOI:** 10.1080/07853890.2021.1964035

**Published:** 2021-10-29

**Authors:** Reza A. Ashrafi, Aila J. Ahola, Milla Rosengård-Bärlund, Tuure Saarinen, Sini Heinonen, Anne Juuti, Pekka Marttinen, Kirsi H. Pietiläinen

**Affiliations:** a Department of Computer Science, Helsinki Institute for Information Technology HIIT, Aalto University, Helsinki, Finland; b Faculty of Medicine, Obesity Research Unit, Research Program for Clinical and Molecular Metabolism, University of Helsinki, Helsinki, Finland; c Folkhälsan Institute of Genetics, Folkhälsan Research Center, Helsinki, Finland; d Department of Nephrology, University of Helsinki and Helsinki University Central Hospital, Helsinki, Finland; e Obesity Center, Endocrinology, Abdominal Center, Helsinki University Hospital and University of Helsinki, Helsinki, Finland; f Department of Gastrointestinal Surgery, Abdominal Center, Helsinki University Hospital and University of Helsinki, Helsinki, Finland

**Keywords:** Bayes’ theorem, computational modelling, dietary intake, one-anastomosis gastric bypass, post-prandial glucose response, Roux-en-Y gastric bypass

## Abstract

**Objectives:**

Our aim was to investigate in a real-life setting the use of machine learning for modelling the postprandial glucose concentrations in morbidly obese patients undergoing Roux-en-Y gastric bypass (RYGB) or one-anastomosis gastric bypass (OAGB).

**Methods:**

As part of the prospective randomized open-label trial (RYSA), data from obese (BMI ≥35 kg/m^2^) non-diabetic adult participants were included. Glucose concentrations, measured with FreeStyle Libre, were recorded over 14 preoperative and 14 postoperative days. During these periods, 3-day food intake was self-reported. A machine learning model was applied to estimate glycaemic responses to the reported carbohydrate intakes before and after the bariatric surgeries.

**Results:**

Altogether, 10 participants underwent RYGB and 7 participants OAGB surgeries. The glucose concentrations and carbohydrate intakes were reduced postoperatively in both groups. The relative time spent in hypoglycaemia increased regardless of the operation (RYGB, from 9.2 to 28.2%; OAGB, from 1.8 to 37.7%). Postoperatively, we observed an increase in the height of the fitted response curve and a reduction in its width, suggesting that the same amount of carbohydrates caused a larger increase in the postprandial glucose response and that the clearance of the meal-derived blood glucose was faster, with no clinically meaningful differences between the surgeries.

**Conclusions:**

A detailed analysis of the glycaemic responses using food diaries has previously been difficult because of the noisy meal data. The utilized machine learning model resolved this by modelling the uncertainty in meal times. Such an approach is likely also applicable in other applications involving dietary data. A marked reduction in overall glycaemia, increase in postprandial glucose response, and rapid glucose clearance from the circulation immediately after surgery are evident after both RYGB and OAGB. Whether nondiabetic individuals would benefit from monitoring the post-surgery hypoglycaemias and the potential to prevent them by dietary means should be investigated.

**KEY MESSAGES**
- The use of a novel machine learning model was applicable for combining patient-reported data and time-series data in this clinical study.
- Marked increase in postprandial glucose concentrations and rapid glucose clearance were observed after both Roux-en-Y gastric bypass and one-anastomosis gastric bypass surgeries.
- Whether nondiabetic individuals would benefit from monitoring the post-surgery hypoglycaemias and the potential to prevent them by dietary means should be investigated.

## Introduction

The prevalence of obesity is increasing at alarming rates and, according to estimates, by year 2030 over 500 million adults worldwide will struggle with obesity [1]. Obesity is a global public health priority [2,3], as it can lead to various physical and metabolic comorbidities and thereby increase the risk of mortality [4,5].

Leading to sustained weight loss and improved survival, bariatric surgery is the most effective treatment of morbid obesity [6,7]. The first paper on gastric bypass was published in the 1960s [8]. Thereafter, several modifications to the bypass techniques have been applied. The laparoscopic Roux-en-Y gastric bypass (RYGB) technique was introduced in 1994 [9]. Since then, the RYGB has been considered a gold standard in bariatric surgery. In 2001, the first series of one anastomosis gastric bypass (OAGB), were published [10]. A number of studies have reported superior weight-loss and diabetes remission related to the OAGB [11–14].

In addition to weight loss, reduced plasma glucose concentrations are observed after gastric bypass procedures [15]. Indeed, higher peak glucose concentrations and more rapid decline to or below basal blood glucose concentration, have been well described [16]. Various phenomena, including improved hepatic and peripheral insulin sensitivity, improved beta-cell glucose sensitivity, increased insulin secretion, reduced basal glucose production and reduced carbohydrate intakes contribute to the improved glycaemia following the operation [15]. Of clinical importance, in individuals with obesity and type 2 diabetes, major long-term improvements in glycaemic control and resolution of diabetes are commonly reported [17,18]. To the best of our knowledge, the acute metabolic effects and the short-term glycaemic responses to the dietary carbohydrate intake, comparing RYGB and OAGB, have not been investigated.

Combining the often noisy dietary data with high-frequency time-series from continuous glucose monitoring devices forms a major statistical challenge. To address this, we adapted a previously published machine learning technique [19], with a time-series model incorporating noisy covariates. An important feature of the model is its ability to correct for the uncertain user-reported meal times, without which the relation between nutrient intake and glucose time-series would be obscured. This allows us to precisely quantify the size of the glucose response in terms of the height and width of the response curve, for a given quantity of carbohydrate intake.

In this paper, our aim was to investigate the use of machine learning for modelling the real-life postprandial glucose concentrations in morbidly obese patients undergoing RYGB or OAGB.

## Methods

**Figure 1.**
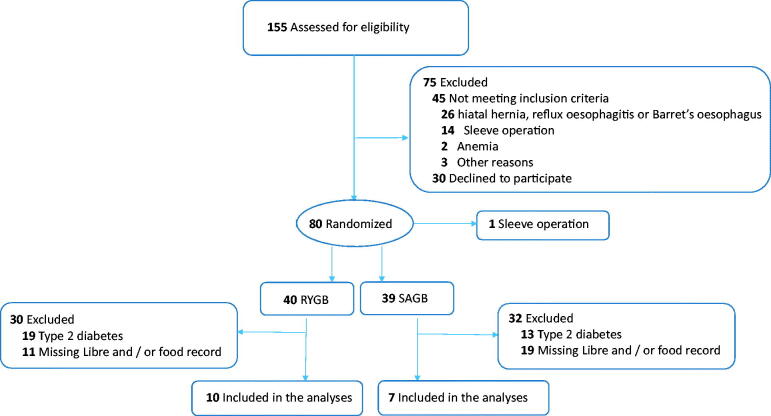
Flowchart of study participants.

This study is part of an open-label prospective randomized controlled RYSA trial (registered in www.clinicaltrials.gov NCT02882685 on 30 August 2016), where patients were randomized to undergo either RYGB or OAGB surgery in one of the two sites in Finland (Helsinki University Hospital and Oulu University Hospital) [20]. The randomization procedures have been previously explained in detail [20]. For investigating the applicability of artificial intelligence for modelling glycaemia, we included data from patients without diabetes operated between November 2016 and April 2019 at Helsinki University Hospital. To be included in the analyses, patients needed to have flash glucose measurements, food diaries, and weight measurements at baseline and 2 weeks postoperatively (Figure 1). The patients operated at Oulu University Hospital were excluded as they did not use the flash glucose measurement devices. The study protocol was approved by the Ethics Committee of Helsinki and Uusimaa Hospital District (HUS/1706/2016) and by Helsinki University Hospital research review board (HUS269/2017). All procedures were conducted in accordance with the 1964 Declaration of Helsinki and its later amendments. Informed consent was obtained from all study participants prior to their inclusion in the study.

### Study participants

Included, in the RYSA trial, were adults (>18 years) with a body mass index (BMI) ≥35 kg/m^2^ who were eligible for gastric bypass surgery according to the treatment guideline [21]. Individuals with anaemia (haemoglobin <120 g/l), endoscopic evidence of hiatal hernia, reflux oesophagitis or Barret’s oesophagus, and those pregnant or lactating were excluded. Excluded were also individuals with any other condition, which could lead to hazard, safety issues and endangering the study procedure, or interfering with the interpretation of the study findings, according to the investigators’ judgement.

### Pre- and postoperative assessments

Patients were assessed at two time points: at baseline (2 months preoperatively at weight stability) and during a two-week period following the operation. At the study visits, participants were thoroughly investigated as previously described [20].

The participants’ weight and height were measured in light clothing and BMI was calculated. Waist circumference was measured at the mid-point between the iliac crest and lowest rib, and the hip circumference was measured at the widest part of the hip. Waist-to-hip ratio was calculated. Fasting blood samples were collected for laboratory analyses of glucose, insulin, HbA_1c_, lipoproteins, triglycerides and high-sensitivity C-reactive protein. Data on medication use and medical history were collected. Following full clinical examination, two weeks prior to the operation, participants were randomized into RYGB or OAGB.

At baseline, participants were weight-stable. However, during 4–6 weeks before the operation (two weeks after baseline), participants were prescribed a very-low energy diet by a nutritionist in order to achieve preoperative weight loss.

At baseline and one day prior to the operation, the participants were provided with the FreeStyle Libre (Abbott Diabetes Care, Maidenhead, UK) flash glucose monitoring devises with instructions for their use. The FreeStyle Libre is a wearable wireless sensor [22], which automatically measures the subcutaneous interstitial fluid glucose concentration at 15-minute intervals. Participants wore the device for two consecutive weeks after the baseline visit and for two consecutive weeks after the operation. Separately for these two periods, we assessed the 3-day time windows for which dietary data were available, to plot the glucose concentrations against carbohydrate intake. In addition, for the entire two-week periods, the complete glucose profiles were available.

Study participants completed a three-day food diary twice. The allocated consecutive days comprised two week-days and one week-end day. The first recording was performed after the baseline visit and the second recording during the second week following the operation, i.e. simultaneously with the FreeStyle Libre measurements. A dietitian gave both oral and written instructions for the patients to complete the diet recording. According to these detailed instructions, participants reported the type, amount and timing of all foods and drinks consumed over the three days. The portion sizes were estimated using household measures. At the time the patients returned the diet recordings, the same dietitian went them through together with the patients to ensure they were complete. Following both operations, an experienced bariatric dietitian advised the patients to initiate a clear liquid meal programme within 24 h of surgery, and over the first two weeks after the surgery, the patients were instructed to progressively transfer to eating soft or creamy foods. The consumption of six to eight small meals (0.5−1 dl every 3 h) was recommended, without drinking beverages at meal times (more than 30 min apart). Additionally, the patients were advised to follow a healthy nutrient-dense diet with 80–130 grams of protein depending on the body weight. Energy and nutrient compositions were calculated using AivoDiet programme (version 2.2.0.1, AIVO, Turku, Finland).

### Surgical procedures

The surgical procedures have been previously described [20]. Briefly, RYGB consisted of a small gastric pouch and 130 cm alimentary and 80 cm biliopancreatic limbs. OAGB consisted of a tubular-shaped gastric pouch created along a 36 Fr bougie and a 210 cm biliopancreatic limb. In both operations the gastrojejunal anastomosis was created with a 45 mm stapler and the remaining anterior defect was hand-sewn.

### Statistical methods

Participant characteristics are presented as means ± standard deviations for normally distributed continuous variables, medians (interquartile ranges) for non-normally distributed continuous variables, and frequencies for categorical variables. Between-group comparisons were conducted with independent samples’ *t*-test, Mann-Whitney *U*-test, and Chi-squared test, in these respective variables. The changes in the times spent in various glycaemic levels were tested using a paired t-test for normally-distributed data. Our model for estimating the glycaemic responses to meal carbohydrates is adapted for challenges in the type of data employed in the study, i.e. it is a robust statistical model capable of rendering replicable and interpretable results from a limited number of noisy observations. The presented model takes into account the uncertainty in the meal timing. This is important in analyses of user-reported data, where reported timings are prone to error, and neglecting associated uncertainties may render to discredited results. The model is fully Bayesian, thus uncertainty of all estimated parameters is characterized with probability distributions. Any prior knowledge can be incorporated in inferring the distribution of the parameters of interest through utilization of the Bayes’ theorem to compute the posterior distribution. The employed hierarchical Bayesian inference model is capable of spontaneously estimating individual level effects, i.e. personalized treatment response, in addition to the population level, while effectively coping with and rectifying measurement errors which are integral in self-reported data. The statistical methods are explained in more detail in Supplementary material.

## Results

Data from 17 participants (8 men, mean ± SD age 47 ± 8 years, age range 28–56 years) were included in the study. Ten (6 men) and 7 (2 men) patients were randomized to RYGB and OAGB, respectively (eTable 1). The two groups were comparable with respect to a number of baseline variables, including BMI, fasting serum lipid and lipoprotein concentrations, HbA_1c_, hsCRP, and fasting glucose and insulin concentrations. Pre- and post-operation weight losses were comparable between the groups. Besides the higher preoperative fat (E%) intake in those randomized to the RYGB, the dietary intakes were also comparable (eTable 2).

Carbohydrate intakes and glucose concentrations reduced significantly in both groups following the operation (eTable 2 and eFigure 1). The maximum glucose concentration decreased in the RYGB group (from 10.0 ± 1.8 to 8.8 ± 0.9 mmol/l, *p* = .042) and the minimum glucose concentration decreased in the OAGB group (from 3.5 ± 0.4 to 2.4 ± 0.3 mmol/l, *p* = .002, eTable 2). Variability of the glucose concentrations were comparable prior and after the operation, in both groups.

**Figure 2.**
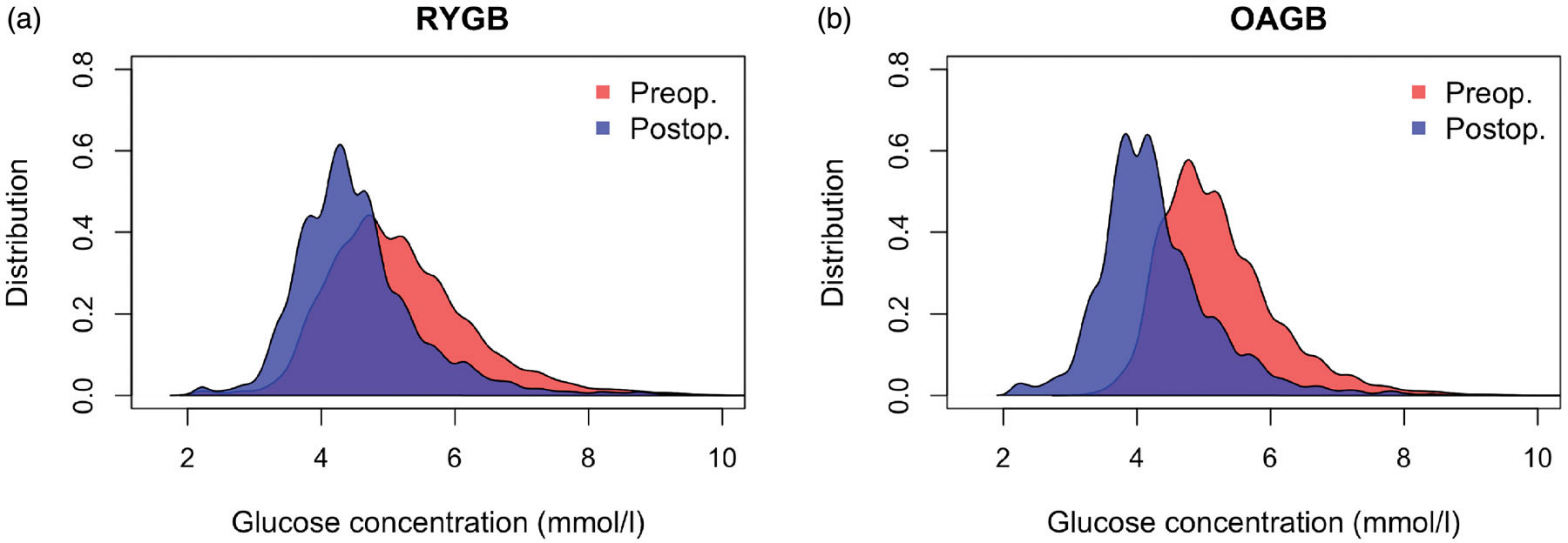
Preoperative and postoperative normalized aggregated histogram of glucose measurements of all individuals in (a) RYGB and in (b) OAGB. RYGB: Roux-en-Y gastric bypass surgery; OAGB: one-anastomosis gastric bypass surgery.

Following the operation, we observed a marked shift in the glucose profile in both groups (Figure 2). In particular, the relative time spent in hypoglycaemia (<4.0 mmol/l) increased in both groups, and the times spent in ≥5 to <6, ≥6 to < 7, and ≥7 mmol/l correspondingly decreased (Table 1). The surgery-induced reduction of the lowest glucose concentration was significantly greater in the OAGB group compared to the RYGB group (from 3.5 ± 0.4 to 2.4 ± 0.3 mmol/l vs. 2.6 ± 0.6 to 2.5 ± 0.4 mmol/l, *p* = .003, Table 1). Other than this, the two groups were comparable.

**Table 1.** A summary of the continuous glucose measurements by the procedure at various stages of the study.

	RYGB			OAGB			RYBG vs. OAGB		
	PreOP	PostOP	*p*	PreOP	PostOP	*p*	*p* ^preOP^	*p* ^postOP^	*p* ^degree of change^
Mean glucose concentration, mmol/l	5.3 ± 0.5	4.5 ± 0.3	<.001	5.3 ± 0.3	4.3 ± 0.3	<.001	.772	.158	.069
Min glucose concentration, mmol/l	2.6 ± 0.6	2.5 ± 0.4	.124	3.5 ± 0.4	2.4 ± 0.3	.002	.002	.630	.003
Max glucose concentration, mmol/l	10.0 ± 1.8	8.8 ± 0.9	.042	9.7 ± 1.9	8.3 ± 1.7	.236	.973	.463	.633
R-Squared, %	18.8 ± 3.4	20.7 ± 3.3	.278	15.4 ± 3.3	19.1 ± 3.3	.099	.068	.321	.541
Time spent in < 4 mmol/l, %	9.2 ± 7.7	28.2 ± 18	.003	1.8 ± 2.2	37.7 ± 19.9	.001	.016	.341	.068
Time spent in > =4 & <5 mmol/l, %	33.9 ± 17.2	46.8 ± 12.8	.186	36.9 ± 20.7	44.6 ± 13.6	.501	.762	.744	.720
Time spent in > =5 & <6 mmol/l, %	34.2 ± 8.8	16.6 ± 5.6	<.001	42.4 ± 13.8	13.5 ± 5.0	<.001	.192	.247	.046
Time spent in > =6 & <7 mmol/l, %	16.2 ± 9.8	5.0 ± 2.3	.006	13.8 ± 7.3	3.1 ± 1.7	.009	.587	.076	.926
Time spent in > =7 mmol/l, %	6.5 ± 5.1	3.4 ± 3.8	.132	4.8 ± 2.5	1.5 ± 2.2	.014	.392	.228	.932

Data are presented as mean ± standard deviation. Between-group comparisons were done with independent samples’ *t*-test, and within-group comparisons before and after the operation were done using paired *t*-test. RYGB: Roux-en-Y gastric bypass; OAGB: one-anastomosis gastric bypass; preOP: prior to the operation; postop: after the operation; BG: blood glucose; CV: coefficient of variation. The times spent in each of the glucose ranges are given as percentages of time relative to the total wear-time of the continuous glucose monitor.

**Figure 3.**
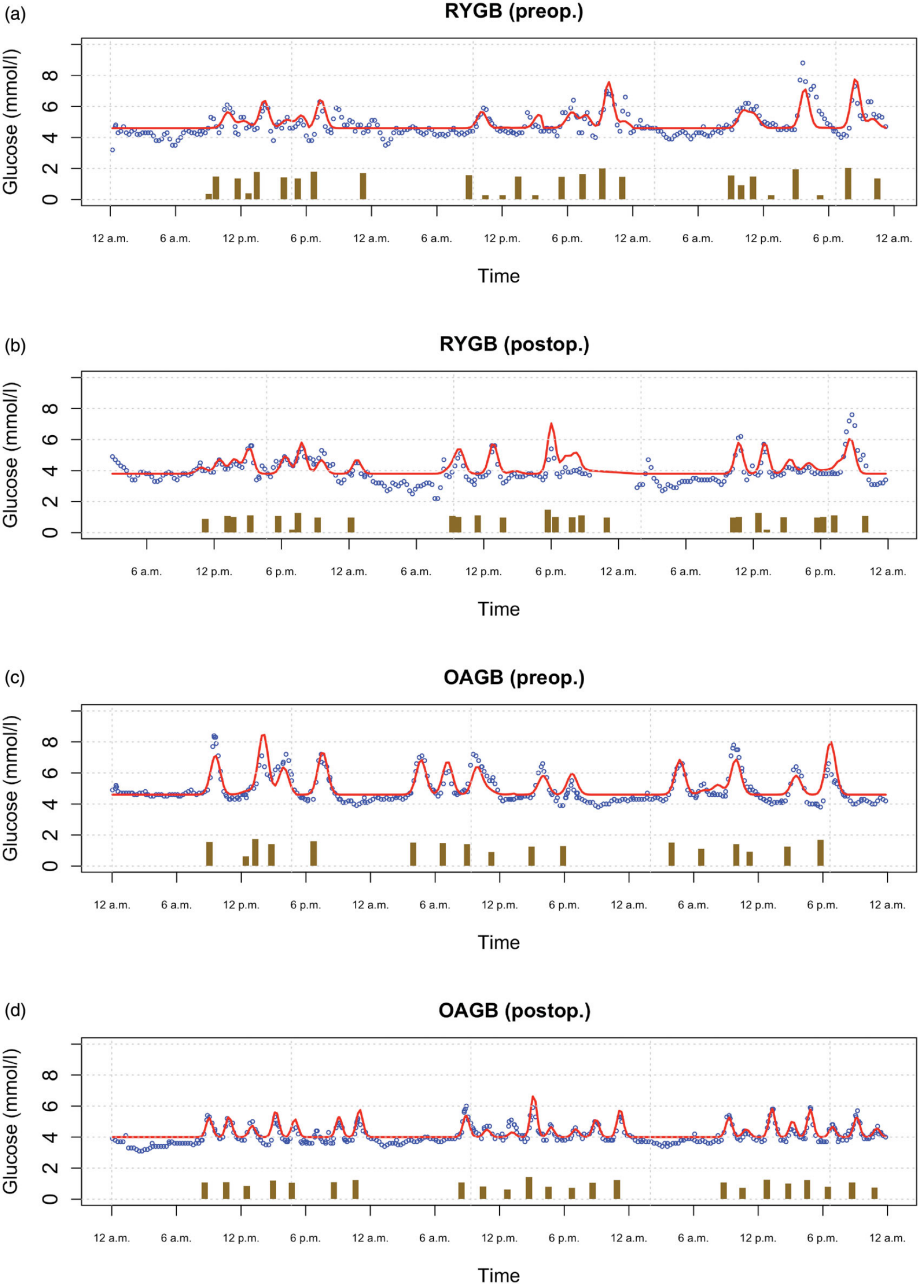
Visualization of the 3-day preoperative time-series for two sample patients before and after the operations: (a) RYGB (preop); (b) RYGB (postop); (c) OAGB (preop); (d) OAGB (postop). Dots are the measurements of glucose concentration. Reported carbohydrate intake, on logarithmic scale, in each meal is indicated by vertical bars. The curves show the fitted models that allow estimating the impact of carbohydrate intake on the glucose level (i.e. how the height and width of the glucose response in the curve depend on the height of the corresponding bar). RYGB: Roux-en-Y gastric bypass surgery; OAGB: one-anastomosis gastric bypass surgery.

**Figure 4.**
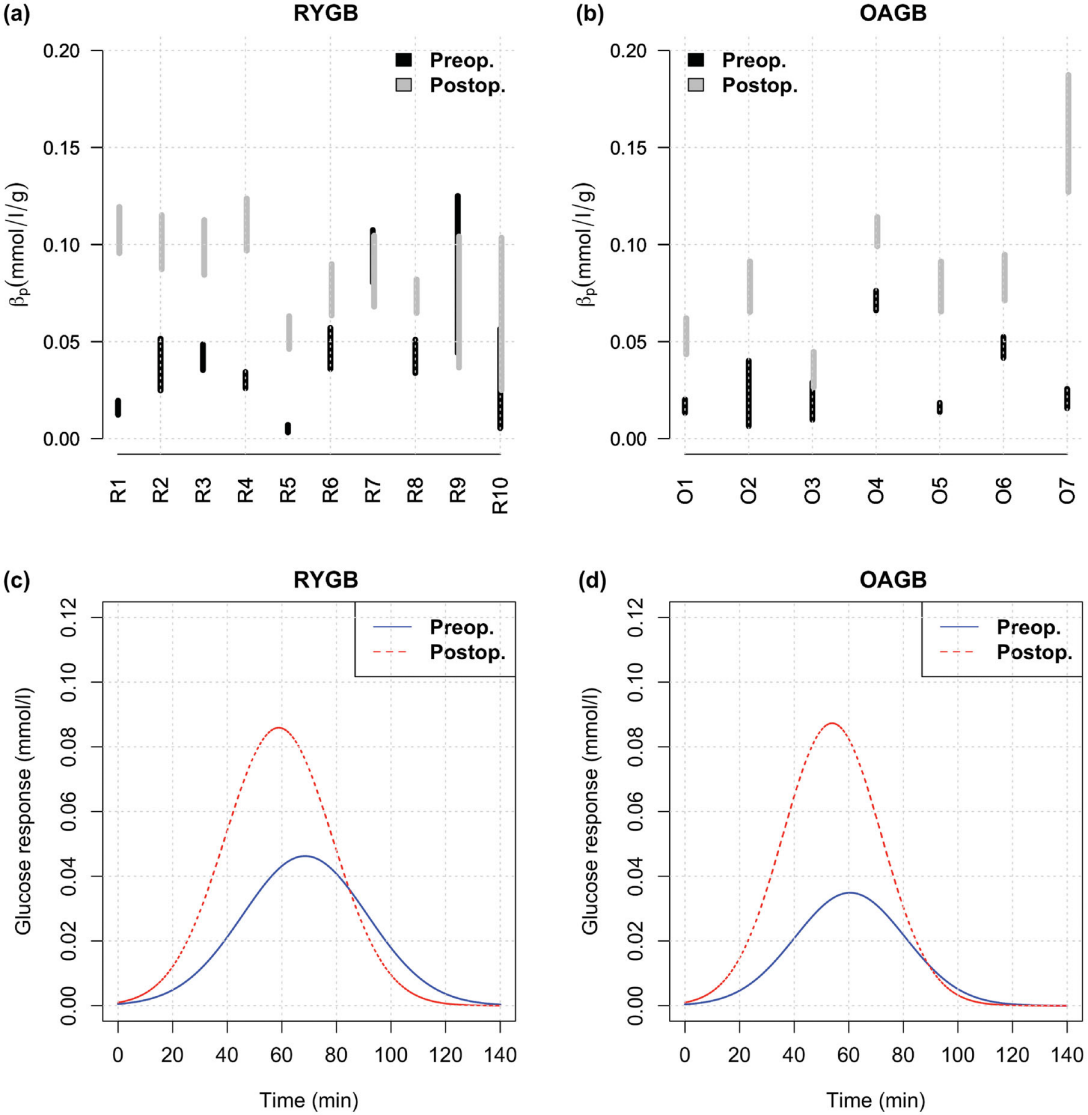
95% confidence interval for the βp parameter for each patient before and after the surgery in (a) RYGB and in (b) OAGB. This parameter indicates the increase in the height of the glucose response curve (the dotted curve in Figure 3) when the amount of dietary carbohydrates increases by one unit (g). Evident in the figure, same amount of carbohydrates causes a higher rise in the glucose concentration in almost all cases after the operation. RYGB: Roux-en-Y gastric bypass surgery; OAGB: one-anastomosis gastric bypass surgery. Average glucose responses to one gram of carbohydrate intake in (c) RYGB and (d) OAGB.

Using artificial intelligence based modelling of the longitudinal data [19], we investigated the glucose responses following the meals. Figure 3 shows the glucose measurements from the FreeStyle Libre (dots), the carbohydrate intakes from each meal (bars), and the fitted response curve (line) that represents the relationship between these two for one example participant from both groups before and after the operations. The response curves are estimated for each patient separately and they allow us to infer the parameter $\beta_{p}$ for each patient. This parameter tells how much the height of the response curve increases postprandially for each gram of carbohydrate. Figure 4(a,b) show the $\beta_{p}$ values separately for each patient in both surgery types. As seen in the figure, the value of the *β*_p_ parameter increased considerably in almost all individuals regardless of the surgery type. Thus, following the operation, the same amount of carbohydrates resulted in a larger increase in the postprandial glucose response. The average parameter values of the estimated response curves, that is the average height parameter of the glucose peak (β) and the average width (duration of the peak) (α) across all cases are summarised in eTable 3. Following the operation, the height of the glucose response increased (increased *β*) but the duration of glucose response was shortened (decreased *α*) in both surgery types. Namely, the mean value of *β* increased from 0.046 to 0.086 mmol/l/g in RYGB, and from 0.034 to 0.088 mmol/l/g in OAGB, following the operation. Furthermore, mean value of *α* (length-scale of the glucose response) decreased from 22.80 to 19.60 min in RYGB, and from 20.56 to 18.33 min in OAGB, postoperatively. The average glucose response to a unit of carbohydrate intake is depicted in Figure 4(c,d), and shows increased height and decreased duration of the response in both surgery types. While the 95% confidence intervals for the difference between these parameters in the two groups are non-overlapping, their values are nevertheless relatively close to each other, indicating that there are no clinically meaningful differences between the two surgeries in their glycaemic responses to meals.

## Discussion

In a sample of morbidly obese individuals undergoing either RYGB or OAGB, using a novel method for estimating the impact of carbohydrate intake on glucose concentration we observed, in a real-life setting, that following the operation the same amount of dietary carbohydrates resulted in a larger increase in the postprandial glucose response. Moreover, while the height of the glucose response increased postoperatively, the duration of the response was significantly shortened. The changes in glucose responses were similar between the two operations, suggesting that in subjects with morbid obesity but without diabetes, both RYGB and OAGB have comparable short-term effects on glycaemia.

To the best of our knowledge, no prior studies have been conducted comparing the acute glucose effects of RYGB and OAGB. In their study, Lazar et al. reported that, in long-term, symptomatic and asymptomatic hypoglycaemias are common observations following both RYGB and OAGB [23]. However, the authors provided no information on the operations. Therefore, it is unknown whether the lengths of the gastric bypasses were different in that study, and whether the operations are thus comparable. Consistent with those observations, however, the time spent in hypoglycaemic levels also increased in the current study. In a retrospective study of obese participants with type 2 diabetes, Almalki et al. reported that compared to RYGB, OAGB resulted in greater remission rate [24]. However, as continuous glucose monitoring was not conducted, no data were available on more acute fluctuations of the blood glucose levels. In the current study, we observed no major differences in acute effects on glucose metabolism between the two operations. Importantly, in both our study and in that of Almalki et al., bypassed limbs were of identical length (80 + 130 cm in RYGB and 210 cm in OAGB). Moreover, in the current study, the gastrojejunostomies were equally wide in both groups. While not different in short-term, the long-term glycaemic effects of the two operation types, using continuous glucose measurements, will need to be assessed in the future.

A number of mechanisms may explain the postoperative changes seen in the glucose responses, including reduction in dietary intake and entering of the less-digested chyme in larger-than-normal quantities into the small intestine and resulting sudden absorption of nutrients [25]. In addition to the observed immediate postprandial increases in the glucose concentrations, it was also evident that the duration of the postprandial glucose peak was significantly shortened, suggesting improved glucose clearance from the circulation. This phenomenon may partly be explained by the functional changes taken place in the remaining small intestine, such as hyperstimulation of the L-cells and subsequent increase in the secretion of glucagon-like peptide-1 (GLP-1), with known glucose-lowering effects due to increased insulin secretion [26], and improved hepatic insulin sensitivity [27].

Considering that the current participants did not have diabetes, the clinical implications related to the changes seen in the glycaemic responses are not known. While it is likely that fast clearance of glucose from the circulation is beneficial, high initial glucose peak has been associated with higher intima-media thickness [28,29], and higher pulse pressure [30], which are known cardiovascular risk factors. On the other hand, the observed reduction in overall glycaemia is likely beneficial. Reduction in glycaemia was accompanied by an increase in time spent in hypoglycaemia after both operations. This increase in hypoglycaemia, related to gastric bypass surgeries, has also previously been reported in other samples of nondiabetic individuals [31]. Importantly, in healthy subjects, hypoglycaemias increase the levels of pro-inflammatory cytokines, markers of lipid peroxidation, reactive oxygen species, and leucocytosis [32]. In individuals with type 2 diabetes with high risk of cardiovascular complications, instead, hypoglycaemic events increased the risk of cardiac arrhythmias, including bradycardia, atrial ectopic activity, and ventricular premature beats [33]. Despite the observed increases in the above risk factors, bariatric surgery has been shown to reduce the risk of cardiovascular events by approximately 50% [34]. It is, therefore, likely that the surgery-associated beneficial changes related to HbA_1c_, weight loss, blood pressure, and concentrations of triglycerides, LDL cholesterol, and HDL cholesterol, overweigh that of daily glycaemic fluctuations [35,36].

The results of this study were made possible by utilizing a machine learning method that explicitly corrected for the noisy user-reported meal times when modelling the time-series from continuous glucose monitoring. In a number of previous studies, factors such as measurement errors related to dietary intake have been addressed [37–39], and in order to improve the quality of the collected dietary data, various statistical approaches have been designed, including the regression calibration [40,41]. On the other hand, the timing uncertainty, accounted for in the current model, has rarely been addressed within this context, while its impact on the accurate estimation of the glucose response may be more dominant [19]. Hence, besides the clinical observations, this study also demonstrated the capability of such a model to yield additional insight into complex data not available by conventional statistical models. Furthermore, the model is likely helpful in applications where the goal is to model time-series data that depend on noisy covariates, for example studies combining patient-reported data with any time-series measurements.

Thorough investigation of the study participants, their randomization into the surgical operations, use of bypass limbs of identical length, modelling the effect of dietary carbohydrate on the glucose curves in response to bariatric surgery, and conducting the study in a real-life setting are important strengths of the study. Although the study sample was small, it is sufficient for investigating the applicability of the novel statistical method used in the current study, as also evidenced by the uniform observations among the study subjects. The reported total energy intake, especially prior to the operations, was low and, indeed, it is well established that individuals with obesity tend to under-report their dietary intake [42]. However, due to a small sample size, we could not afford to exclude any participants based on their reported energy intake. Regardless of the potential underreporting, distinct differences in glycaemic responses before and after the operations were observed, suggesting that the model worked well using the collected data. Moreover, while it is acknowledged that also other dietary constituents, such as fibres, glycaemic index, fats, and proteins impact the postprandial glycaemic fate, only carbohydrate intake was taken into account, in the model. In principle, including additional covariates is straightforward. In practice, however, this leads to some challenges, e.g. unsatisfactory convergence of the Markov chain Monte Carlo algorithm [43] used for model fitting, nonidentifiability of the model with correlated covariates, and consequent lack of interpretability of the results. Given that carbohydrate intake is a well-known causative dietary factor for the glucose response, simplifying the model accordingly ensures the robustness of the findings. Another simplifying assumption is that the model uses a constant response baseline, i.e. glucose measurements between meal times. While this is not physiologically fully accurate, it nevertheless gives a reasonable fit to the data (Figure 3). In addition, our aim was to estimate the causal effect of carbohydrate intake on the preoperative and postoperative glucose responses. Using a varying baseline (e.g. a slow varying Gaussian process regression [19], would make it difficult to separate the variation in the baseline from the variation caused by the actual input (food). Thus, employing a simple constant baseline enables the glucose response to the carbohydrate intake to be estimated. Further improvements of these aspects are within our planned future research. Finally, due to the exclusion of individuals with diabetes, the results are not confounded by diabetes medication or glucose toxicity. Subsequently, our observations may not be directly applied to those with diabetes.

In conclusion, in this study we demonstrated the utility of a novel technique in a clinical study that combined patient-reported and time-series data. In these analyses, marked increase in postprandial glucose concentrations, rapid clearance of glucose from the circulation, and increase in time spent in hypoglycaemia were observed after both RYGB and OAGB, with no clinically significant differences between groups. Whether nondiabetic individuals undergoing gastric bypass surgeries would benefit from monitoring the post-surgery hypoglycaemias and the potential to prevent them by dietary means should be investigated.

## Supplementary Material

Supplemental Material

## Data Availability

According to the General Data Protection Regulation of the European Union (679/2016), the principles of data protection should apply to any information concerning an identified or identifiable natural person and that personal data which have undergone pseudonymization, which could be attributed to a natural person by the use of additional information should be considered to be information on an identifiable natural person. Thus, according to the GDPR, all pseudonymized data are considered personal data and cannot be published openly. Therefore, we are bound to the law and to the strict hospital policies, and are unable to share the data. However, the institutional (Helsinki and Uusimaa Hospital District) contact details for potential future data requests are as follows: https://huspalvelu.microsoftcrmportals.com/fi-FI/.
