# Supplementary material for: Computational modelling of self-reported dietary carbohydrate intake on glucose concentrations in patients undergoing Roux-en-Y gastric bypass versus one-anastomosis gastric bypass
Source: Ann Med. 2021 Oct 29;53(1):1885–95. doi: 10.1080/07853890.2021.1964035 (PMC8567939; doi:10.1080/07853890.2021.1964035)
Supplement: Supplemental Material [file IANN_A_1964035_SM4041.pdf]

The impact of bariatric surgery on the postprandial glucose response to meal carbohydrate

Reza A. Ashrafi, PhD[a†], Aila J. Ahola, PhD[b,c,d†], Milla Rosengård-Bärlund, MD, PhD[e], Tuure Saarinen, MD, PhD.p.s.[b,f], Sini Heinonen, MD, PhD[b], Anne Juuti, MD, PhD[f], Pekka Marttinen, PhD[a], Kirsi H. Pietiläinen, MD, PhD[b,e*]

[a] Helsinki Institute for Information Technology HIIT, Department of Computer Science, Aalto University, Finland; [b] Obesity Research Unit, Research Program for Clinical and Molecular Metabolism, Faculty of Medicine, University of Helsinki, Finland; [c] Folkhälsan Institute of Genetics, Folkhälsan Research Center, Helsinki, Finland; [d] Department of Nephrology, University of Helsinki and Helsinki University Central Hospital, Helsinki, Finland; [e] Obesity Center, Endocrinology, Abdominal Center, Helsinki University Hospital and University of Helsinki, Helsinki, Finland; [f] Department of Gastrointestinal Surgery, Abdominal Center, Helsinki University Hospital and University of Helsinki, Finland; [†] Authors with equal contribution

***Correspondence to** Kirsi Pietiläinen, Obesity Research Unit, Biomedicum 1, Haartmaninkatu 8, PO Box 63, FI-00014 University of Helsinki, Finland. Tel +358 505992295. Email: kirsi.pietilainen@helsinki.fi

Contents of the supplementary material

1. Detailed description of the statistical methods

2. eTable 1. Baseline characteristics of the study population by the procedure, as well as weight, BMI, and weight loss at various stages of the study

3. eTable 2. Mean energy, macronutrient and fibre intakes by the procedure at various stages of the study

4. eTable 3. Parameter values representing the impact of carbohydrate intake on the height and the width parameters of the glucose response

4. eFigure 1. Scatter plots of 3-day median glucose concentration vs. 3-day mean reported daily carbohydrate intake

Detailed description of the statistical methods

In Bayesian inference parameters of interest are considered as random variables instead of unknown constants, and thus any prior knowledge can be incorporated in inferring the distribution of the parameters of interest through utilization of the Bayes' theorem to compute the posterior distribution. Therefore, Bayesian statistics provides more intuitive, meaningful, and interpretable inferences on complex problems through utilization of all available information. The method is designed as follows.

Collected glucose concentration data of the pth patient $p \in 1,2,\dots,P$, where P is the total number of patients, is a time series vector of length N_p i.e.,

$$\boldsymbol{y_p} = \left(\boldsymbol{y_{p1}},\dots,\boldsymbol{y_{pN_p}}\right)^{\mathbf{T}}$$

Furthermore, each patient has M_p recorded meals, indexed by $m \in 1,2,\dots,M_p$

$$\boldsymbol{x_p} = (\boldsymbol{x_{p1}},\dots,\boldsymbol{x_{pM_p}})^{\mathbf{T}} \text{ for all } p.$$

Here, $\boldsymbol{x_{pM_p}}$ is the amount of carbohydrates in grams in the mth meal of the pth patient, calculated as the sum of sugar and starch. We assumed that the glucose curve of an individual p, $\boldsymbol{y_p}$, is given by

$$\mathbf{y}_p = T_p + \textstyle\sum_m R_{pm} + \mathbf{e},$$

where $T_p \in \mathbb{R}$ is a constant baseline which we set to the median of the observations, $R_{pm} \in \mathbb{R}^P$ is the additive glucose response to the mth meal, and $e = \left(e_1,\dots,e_{N_p}\right)^T$ is a vector of Gaussian observation errors. In words, the glucose curve equals the sum of the trend and meal-specific response curves, plus noise.

The response function specifies the impact a meal has on the glucose curve over the time. Here we assume a symmetric bell-shaped curve as the response function, which is expressed by two parameters, patient and meal-specific h_{pm} and patient-specific α_p. Here, h_{pm} describes the height of the glucose response, while α_p is the length-scale, and therefore proportionally represents the duration of the glucose peak. The total duration of the response is approximately 5 α_p.

To estimate how the glucose response depends on the amount of carbohydrates in a meal, we allowed the height of the response, h_{pm}, to depend on the amount of carbohydrates:

$$\mathrm{h}_{pm} = \beta_{\mathrm{p}}\, \boldsymbol{x}_{pm}.$$

In the above equation, the coefficient β_p represents the *personalized impact* of the carbohydrate intake on the height of the response for the pth individual. In other words, β_p shows the amount of glucose response peak, if one gram of carbohydrate is consumed. To efficiently perform the statistical inference with the limited data available, we introduced a Bayesian hierarchical prior,[1] which enables information to be shared across individuals. Further details of the method and of the prior distribution are given in the original article.[2]

Statistical inference was done using Markov chain Monte Carlo (MCMC) algorithm implemented in software STAN.[3] We also rely on a Bayesian t-test,[4] when assessing the significance of a difference between estimated posterior distributions, i.e. estimated distribution of preoperative and postoperative average height parameter β and length- scale parameter α. It generates the posterior distribution of the difference between the means of the two groups and is considered significant with 5% level of significance if the 95% highest density interval (HDI) does not contain the value zero, in which case the null hypothesis is rejected.

eTable 1 Baseline characteristics of the study population by the procedure, as well as weight, BMI, and weight loss at various stages of the study

	RYGB (n = 10)		OAGB (n = 7)		
		min-max		min-max	p
**Baseline characteristics**					
Men, n	6		2		0.335
Age, years	50 ± 8	32 – 56	43 ± 7	28 – 49	0.072
Total cholesterol, mmol/l	4.4 (4.1 – 4.9)	2.7 – 8.8	4.5 (4.1 – 5.5)	3.9 – 5.8	0.601
HDL-cholesterol, mmol/l	1.3 ± 0.3	0.9 – 1.7	1.2 ± 0.1	1.0 – 1.4	0.434
LDL-cholesterol, mmol/l	2.9 ± 0.9	1.5 – 4.7	3.4 ± 0.7	2.6 – 4.6	0.202
Triglycerides, mmol/l	1.2 (0.9 – 1.5)	0.7 – 10.1	1.0 (0.9 – 1.5)	0.7 – 1.5	0.364
hsCRP, mg/l	4.6 ± 4.4	0.7 – 13.6	3.0 ± 2.1	0.7 – 6.6	0.364
HbA_{1c}, mmol/mol	35 (31 – 39)	29 – 40	34 (33 – 35)	24 – 40	0.813
Insulin, mU/l	14.6 (10.0 – 26.3)	5.5 – 48.7	24.5 (16.5 – 55.0)	14.3 – 126.2	0.142
Fasting glucose, mmol/l	5.8 ± 0.5	5.2 – 6.7	5.8 ± 0.5	5.3 – 6.7	0.987
Waist circumference, cm	126 (123 – 141)	116 – 149	120 (104 – 133)	101 – 136	0.283
Hip circumference, cm	125 (118 – 150)	110 – 158	125 (117 – 134)	115 – 135	0.683
WHR	1.00 (0.87 – 1.14)	0.83 – 1.20	0.90 (0.88 – 1.07)	0.87 – 1.12	0.683
**Weight , BMI, and weight loss**					
Weight at BL, kg	127.2 ± 14.0	107.3 – 145.0	125.8 ± 16.8	108.3 – 144.7	0.850
Weight at OP day, kg	122.4 ± 12.6	106.3 – 140.4	122.6 ± 17.4	104.0 – 142.2	0.975
Weight 2-3 weeks PostOP, kg	115.3 ± 12.0	97.5 – 131.0	113.5 ± 15.6	98.9 – 130.6	0.794
BMI at BL, kg/m^2	42.9 ± 5.3	35.0 – 49.9	43.9 ± 3.6	39.5 – 50.7	0.664
BMI at OP day, kg/m^2	41.1 ± 5.2	33.0 – 47.6	42.9 ± 4.2	37.4 – 50.4	0.476
BMI 2-3 weeks PostOP, kg/m^2	38.9 ± 5.2	30.0 – 45.7	39.7 ± 3.9	33.3 – 46.2	0.708
PreOP weight change (BL vs. OP day), %	-3.7 ± 2.5	-5.7 – 2.1	-2.6 ± 2.1	-5.6 – 0	0.353
PostOP weight change (OP day vs PostOP), %	-5.8 ± 1.5	-8.9 – -4.1	-7.3 ± 2.2	-10.9 – -4.9	0.107

Frequency is shown for categorical variables, mean ± standard deviation for continuous variables with a normal, and median (interquartile range) for continuous variables with a non-normal distribution. Between-group comparisons were done with Chi-squared test, independent samples' t-test, and Mann-Whitney U-test, respectively. BMI, body mass index; RYGB, Roux-en-Y gastric bypass surgery; OAGB, one-anastomosis gastric bypass surgery; hsCRP, high-sensitivity C-reactive protein; WHR, waist-to-hip ratio; BL, baseline; OP, operation; PostOP, after operation; PreOP, before operation.

eTable 2 Mean energy, macronutrient and fibre intakes by the procedure at various stages of the study

	RYGB			OAGB			RYGB vs. OAGB	
	PreOP	PostOP	P	PreOP	PostOP	P	P^{preOP}	P^{postOP}
Energy, kJ	8960 ± 4310	4277 ± 881	0.005	8018 ± 1849	4186 ± 1079	0.007	0.597	0.850
Energy, kcal	2139 ± 1030	1021 ± 210	0.005	1915 ± 442	1000 ± 258	0.007	0.598	0.853
Carbohydrates, g	214 ± 146	99 ± 23	0.025	203 ± 71	99 ± 33	0.024	0.864	0.964
Carbohydrates, E%	38.3 ± 8.5	38.9 ± 5.9	0.850	42.1 ± 7.5	40.7 ± 11.8	0.763	0.366	0.682
Fats, g	92 ± 44	36 ± 12	0.001	67 ± 25	32 ± 18	0.009	0.184	0.629
Fats, E%	39.0 ± 5.1	31.0 ± 6.0	0.018	30.6 ± 8.2	28.8 ± 10.8	0.671	0.020	0.593
Proteins, g	99 ± 26	69 ± 17	0.011	106 ± 18	70 ± 28	0.006	0.530	0.936
Proteins, E%	19.5 ± 3.8	27.1 ± 5.1	<0.001	23.1 ± 6.5	27.2 ± 6.4	0.109	0.176	0.985
Alcohol, g	1.6 ± 3.9	0.7 ± 2.2	0.495	3.7 ± 9.9	0	0.356	0.546	0.420
Alcohol, E%	0.6 ± 1.4	0.4 ± 1.4	0.772	1.3 ± 3.4	0	0.356	0.570	0.420
Fibre, g	20 ± 5	9 ± 3	<0.001	22 ± 7	8 ± 3	0.006	0.532	0.829

Data are presented as means ± standard deviations. Between-group comparisons were done with independent samples' t-test, and within-group comparisons before and after the operation were done using paired t-test. RYGB, Roux-en-Y gastic bypass surgery; OAGB, one-anastomosis gastric bypass surgery; PreOP, prior to the operation; PostOP, after the operation; E%, percentage of total energy intake.

eTable 3 Parameter values representing the impact of carbohydrate intake on the height and the width parameters of the glucose response

	β (mmol/l/g)				α (minutes)			
	**PreOP**	**PostOP**	**P**	**Difference**	**PreOP**	**PostOP**	**P**	**Difference**
RYGB	0.046 ± 0.016	0.086 ± 0.017	<0.001	0.038, 0.040	22.80 ± 3.35	19.60 ± 3.36	<0.001	-4.05, -3.20
OAGB	0.034 ± 0.016	0.088 ± 0.018	<0.001	0.053, 0.054	20.56 ± 4.49	18.33 ± 3.64	<0.001	-2.38, -2.04

Data are presented as mean ± standard deviation. Within-group comparisons before and after the operation were done with the Bayesian t-test. β, height parameter of the glucose response; α, width parameter of the glucose response; PreOP, prior to the operation; PostOP, after the operation; Difference, the 95% CI for the difference between parameters after and before the surgery; RYGB, roux-en-Y gastric bypass; OAGB, one-anastomosis gastric bypass.

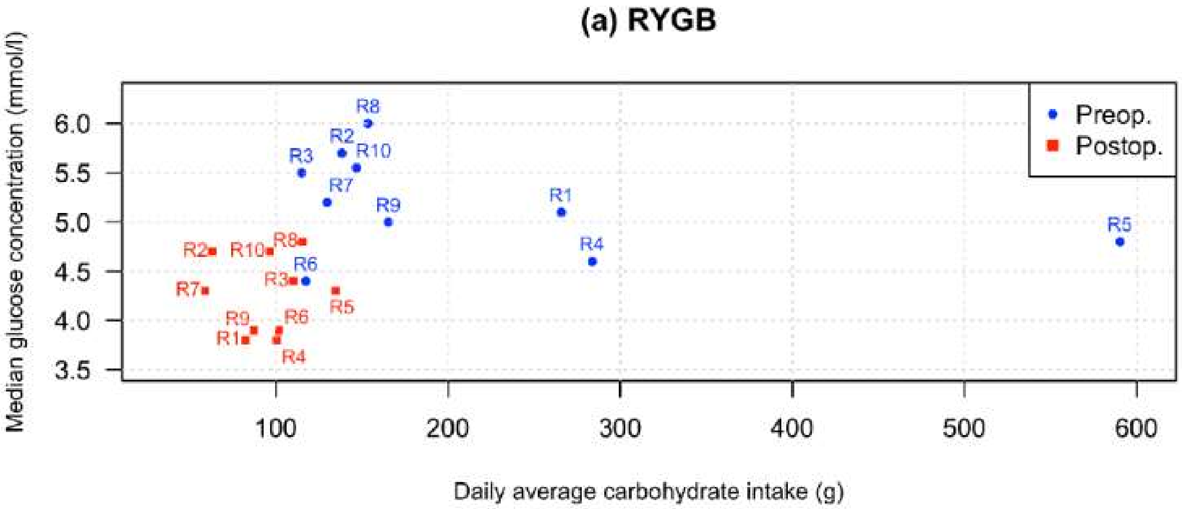

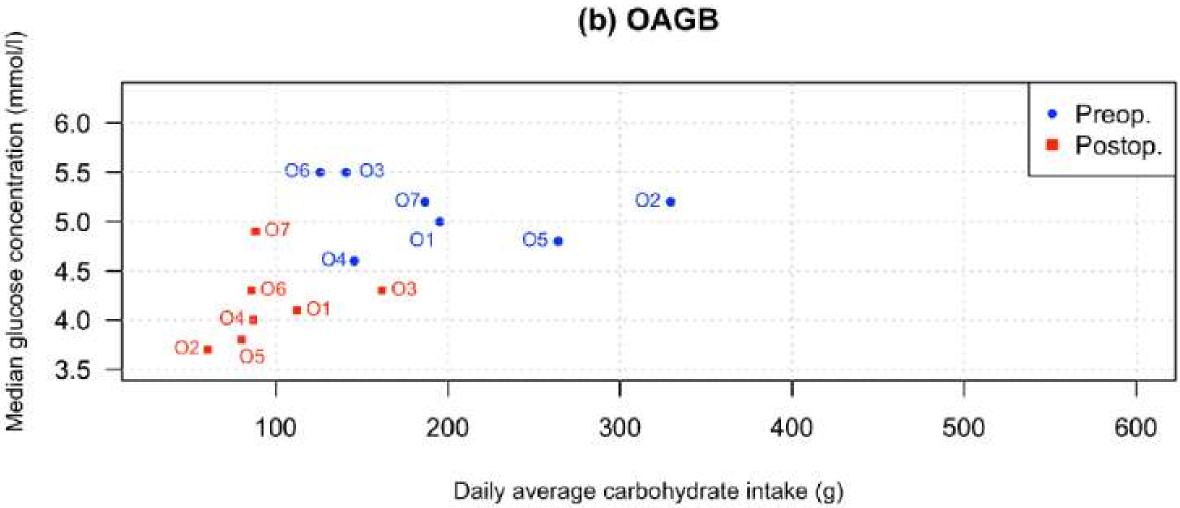

eFigure 1. Scatter plots of 3-day median glucose concentration vs. 3-day mean reported daily carbohydrate intake in a. RYGB and in b. OAGB. Both preoperative and postoperative values are calculated over the respective three-day measurement periods and both values are reported for each individual. RYGB, Roux-en-Y gastric bypass surgery; OAGB, one-anastomosis gastric bypass surgery.

References

1. Gelman A, Stern HS, Carlin JB, et al. Bayesian data analysis. Chapman and Hall/CRC. 2013.

2. Zhang G, Ashrafi RA, Juuti A, Pietiläinen K, Marttinen P. Errors-in-variables modeling of personalized treatment-response trajectories. *IEEE J Biomed Health Inform.* 2021;25(1):201-208.

3. Carpenter B, Gelman A, Hoffman M, et al. Stan: A Probabilistic Programming Language. *Journal of Statistical Software.* 2017;76:1–32.

4. Kruschke JK. Bayesian estimation supersedes the t test. *J Exp Psychol Gen.* 2013;142:573-603.
